# ASAP3 regulates microvilli structure in parietal cells and presents intervention target for gastric acidity

**DOI:** 10.1038/sigtrans.2017.3

**Published:** 2017-02-24

**Authors:** Jin Qian, Yueyuan Li, Han Yao, Haiying Tian, Huanbin Wang, Luoyan Ai, Yuanhong Xie, Yujie Bao, Lunxi Liang, Ye Hu, Yao Zhang, Jilin Wang, Chushu Li, Jiayin Tang, Yingxuan Chen, Jie Xu, Jing-Yuan Fang

**Affiliations:** 1 Division of Gastroenterology and Hepatology, Key Laboratory of Gastroenterology and Hepatology, Ministry of Health, State Key Laboratory for Oncogenes and Related Genes, Renji Hospital, School of Medicine, Shanghai Jiao Tong University, Shanghai Institute of Digestive Disease, Shanghai, China

## Abstract

Gastric acidity-associated disorders such as peptic ulcer and reflux diseases are widespread, and the reported resistance and side effects of currently used medicines suggest an urgent requirement for alternative therapeutic approaches. Here we demonstrate a critical role of ASAP3 in regulating the microvilli structure of parietal cells *in vivo*, and reveal the feasibility of controlling gastric acidity by targeting ASAP3. Conditional knockout of ASAP3 in mice caused elongation and stacking of microvilli in parietal cells, and substantially decreased gastric acid secretion. These were associated with active assembly of F-actin caused by a higher level of GTP-bound Arf6 GTPase. Consistently, a small molecular compound QS11 inhibited ASAP3 function and significantly reduced gastric acidity *in vivo*. Of note, the expression of ASAP3 was positively correlated with gastric acid secretion in 90 human cases, and high expression of ASAP3 was associated with reflux disease and peptic ulcer. These results reveal for the first time that ASAP3 regulates the microvilli structures in parietal cells. Our data also suggest ASAP3 as a feasible and drugable therapeutic target for gastric acidity-associated diseases.

## INTRODUCTION

Gastric acid secretion is required for the digestion of protein, absorption of vitamin B12, iron and calcium,^1^ as well as the prevention of bacterial overgrowth. Insufficient gastric acidity may increase the risks of osteoporosis,^2^ rheumatoid arthritis,^3^ infectious diseases^4^ and malignancy,^5^ whereas excess acid secretion may also lead to widespread diseases such as peptic ulcer^6^ and gastro-esophageal reflux disease (GERD).^7–10^ Drugs that control gastric acid secretion, such as proton pump inhibitors (PPIs), are used in the treatment of many conditions such as dyspepsia, peptic ulcer, GERD, Barrett’s esophagus, eosinophilic esophagitis and so on. The PPIs are among the most widely sold drugs in the world and are in general well tolerated, but resistance and adverse effects such headache, nausea, diarrhea, abdominal pain, fatigue and dizziness have been reported.^11^ Thus, there is a need for discovering alternative approaches for controlling gastric acidity, which may provide therapeutic opportunity for patients who have unsatisfactory response to PPIs.

The gastric parietal cells contain extensive apical membrane on the surface of microvilli, constituting a structural basis for transient proton pumping. Currently, it is poorly understood how the structure of microvilli is regulated. Several *in vivo* studies have suggested the roles of Arf6,^12^ Hip1r^13^ and ezrin^14,15^ in regulating gastric acid secretion, but these were thought to function through the vesicle transportation pathway. Similarly, an *in vitro* study based on isolated rabbit parietal cells indicated that ASAP3, an Arf GTPase activating protein, may affect gastric acid secretion by binding ezrin and inducing the fusion of tubulovesicles to apical membrane.^16^

**Figure 1:**
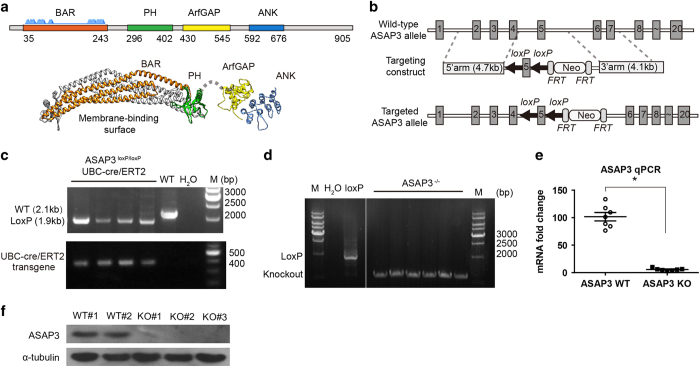
Disruption of the *ASAP3* gene. (**a**) The ASAP3 encodes a multi-domain protein including the BAR, PH, ArfGAP and ANK domains. The BAR and PH domains bind stably to form a homodimer with curved structure, according to homology modeling based on ASAP1 (4nsw.pdb). The ArfGAP and ANK domains of ASAP3 form a stable complex while binding to Arf6 (3lvq.pdb). (**b**) The mouse ASAP3 genomic locus (top) and the vector (middle) designed to replace exon 5, resulting in the ASAP3^loxP^ allele in embryonic stem cells (bottom). (**c**) The ASAP3^loxP^ mice were crossed to UBC-cre/ERT2 mice to obtain litters carrying homozygous ASAP3^loxP/loxP^ and the UBC-cre/ERT2 transgene. Polymerase chain reaction (PCR) genotyping of tail DNA from mice showing a 2.1-kb and a 1.9-kb fragment corresponding to the wild-type (WT) and loxP alleles, respectively (top). The UBC-cre/ERT2 transgene in these mice was also confirmed by PCR (bottom). (**d**) PCR validation of ASAP3 knockout by tamoxifen induction. The genomic DNA of ASAP3^loxP/loxP^ and tamoxifen-induced mice were isolated and amplified using specific primers binding to the recombination site. The single amplicon size for each condition indicated the homozygous status of ASAP3^loxP/loxP^ and ASAP3^−/−^ mice. (**e**) The messenger RNA (mRNA) level of ASAP3 in ASAP3^loxP/loxP^-UBC-cre/ERT2 mice which were induced with tamoxifen was barely detected by quantitative PCR (qPCR), compared to that in ASAP3^WT^ mice (*n*=7 per genotype). Data (mean±s.e.m.) were normalized to 18S expression in the same samples and reported as fold change relative to WT. (*P<0.01, Student’s *t*-test). (**f**) Western Blot showing disrupted expression of ASAP3 in ASAP3^loxP/loxP^-UBC-cre/ERT2 mice that were induced with tamoxifen, but not in ASAP3^WT^ mice (top). The expression of α-tubulin was also detected as control (bottom). The data are representative of immunoblots from seven littermates per loading group.

ASAP3 contains the BAR (Bin, Amphiphysin and Rvs) and PH domains in its amino portion and ArfGAP and ANK (ankryin repeat) domains in its carboxy portion (Figure 1a). The PH domain of ACAP1 (a homolog to ASAP3) functions together with the BAR domain to bind membrane and generate curvature.^17^ The ArfGAP and ANK domains of ASAP3 have been found to bind Arf6.^18^ These structural domains indicate that ASAP3 may affect Arf6-dependent actin assembly near the curved apical membrane, supporting a potential role in regulating microvilli structure.

In order to investigate the roles of ASAP3 in parietal cells *in vivo*, we generated a conditional knockout mouse model and applied a small molecular compound QS11 to suppress the function of ASAP3. The structural and functional alterations of ASAP3^−/−^ parietal cells were studied in both resting and stimulated phases, and the long-term effect of ASAP3 disruption was examined by histopathology. The correlation between ASAP3 expression and gastric acidity was analyzed in 90 human cases. By these efforts, we aim to reveal the roles of ASAP3 in gastric acid secretion and clarify its therapeutic significance for gastric acidity-associated disorders.

## MATERIALS AND METHODS

### Ethics statement

The protocol had the approval of the Ethics Committee of the Shanghai Jiao-Tong University School of Medicine, Renji Hospital, and the research was carried out according to the provisions of the Helsinki Declaration of 1975. Written informed consent was obtained from all participants involved in the study, and clinical information was collected with Institutional Review Board approval.

### Patient samples

Samples and gastric content were analyzed in patients who underwent gastroscopy in the Division of Gastroenterology and Hepatology, Shanghai Renji Hospital during 08:00–10:00 hours after fasted over 12 h. Patients with bile reflux, history of gastrointestinal tumor or surgery, or administration of PPIs or non-steroidal anti-inflammatory drugs were excluded for analysis. Gastric mucosal tissue specimens and gastric fluid were from 90 patients who underwent gastric endoscopy at the Shanghai Renji Hospital from 2013 to 2014. Gastric and duodenal ulcers were diagnosed by endoscopic examination. GERD was diagnosed by endoscopy combined with the Gastroesophageal Reflux Disease Questionnaire (GerdQ).^19^ The rate of GERD diagnosed (44/90) was related to the fact that all patients who underwent gastroscopy reported certain discomfort.

For detection of gastric acidity, the gastric contents collected by endoscopy was centrifuged at 500 *g* for 5 min, and the supernatant was recovered for measuring pH and titrating to pH 7.0 with 0.01 mol l^−1^ NaOH. Practically, it is difficult to acquire all the gastric fluid in the gastroscopic examination (factors include the capacity of collecting container, residue amount in the sampling duct and so on), thus the concentration of H^+^ in the gastric fluid sample was used to represent the gastric acidity.

### Immunohistochemistry of patient tissue samples

Gastric body biopsy were routinely formalin-fixed, paraffin embedded and cut into 4-μm-thick sections then placed on polylysine-coated slides. After incubated in a 60 °C oven for 60 min, the slides were deparaffinized in xylene and dehydrated with graded ethanol, then washing with distilled water and phosphate-buffered saline. Endogenous peroxidase activity was blocked using 3.0% hydrogen peroxide for 15 min at room temperature. For antigen retrieval, the slides were placed in citrate antigen-repairing solution and heated in a high-pressure cooker in the gastric fluid sample was used to represent the gastric acidity. Immunohistochemistry of patient tissue samples until steam arose and then cooled off at room temperature for 40 min. After phosphate-buffered saline washing, the sections were blocked in goat serum for 30 min, then incubated with rat monoclonal antibody for human ASAP3 (1:30 dilution; sc-365840, Santa Cruz Biotechnology) overnight at 4 °C. The slides were washed with phosphate-buffered saline and incubated with universal (anti-mouse/rabbit) detection reagent (horseradish peroxidase; D-3004, Supervision, Changdao Biotech Co., Shanghai, China) as second antibody for 40 min at room temperature. After washing with phosphate-buffered saline, the immune reaction was demonstrated with 3,3'-Diaminobenzidine. The sections were then counterstained with hematoxylin, dehydrated and mounted. Tissue slides were visualized using an Olympus BX43F microscope and figures were captured using Olympus DP26 digital camera. A total of three fields selected from hot-spot areas (×400 objective lens) were acquired per slide. The integral optic density, representing the ASAP3 level of parietal cells in the field, and area of interest were counted and measured using Image-Pro Plus 6.0 software (Media Cybernetics Inc., Rockville, MD, USA). Thus the optic density (integral optic density/area of interest) represented the concentration of ASAP3 in each gastric body sample.

### Generation of ASAP3-deficient mice

The ASAP3 targeting vector bearing a gene-trap cassette doubly flanked by LoxP and FRT was constructed using the method described previously.^20^ A 4.7 kb fragment corresponding to the exons 2–4 of ASAP3 (EnsemblGene ID: ENSMUST00000047526) was cloned as the 5′ arm, followed by *loxP*-flanked exon 5 and FRT-neo-FRT site. The 4.1-kb-long 3′ arm overlapped with exons 6 and 7. Murine embryonic stem cells were electroporated with the linearized targeting vector and screened for homologous recombination by 300 μg ml^−1^ G418 and 2 μm ganciclovir for 8 days. Two correctly targeted embryonic stem clones were selected, expanded and injected into C57BL/6 mouse blastocysts to generate chimeric mice that were bred to obtain germ-line transmission. The experiments described in this study were performed using ASAP3 wild-type and knockout littermates on a C57BL/6 X 129SV mixed background in Shanghai Research Center for Model Organisms. The resultant ASAP3^loxP/loxP^ mice were then crossed with UBC-cre/ERT2 mice, and adult offspring were induced by sequential peritoneal injection of tamoxifen for 7 days to knockout ASAP3. Animal procedures were approved by the Institutional Animal Care and Use Committees at Shanghai Research Center for Model Organisms and the Renji Hospital of Shanghai Jiao Tong University Medical School.

### Immunofluorescence of mouse tissue samples

Mouse tissue sections were deparaffinized in xylene and rehydrated using a graded series of ethanol. All slides were treated with NaBH4 to suppress autofluorescence of tissues. The expression and localization of ASAP3, H^+^-K^+^-ATPase, ezrin, F-actin, Arf6, calcium pump pan PMCA ATPase and Ki67 were probed with the primary antibodies (dilution 1:50), followed by labeling with secondary antibodies (Alexa488-anti-rabbit or Alexa546-anti-mouse). After staining with DAPI (1:10 000), the coverslips were added with anti-fade reagent (ProLong Gold, Invitrogen, Carlsbad, CA, USA) and kept in the dark for 24 h. Images were acquired with a confocal fluorescence microscope (LSM710, Carl Zeiss). The colocalization analyses were performed using the microscopy software package Zen (Carl Zeiss) and ImageJ. Intensity profiles were analyzed and displayed using the ImageJ software package (More experimental procedures and statistical analysis are described in Supplementary Information).

## RESULTS

### Disruption of the *ASAP3* gene

First, we found that ASAP3 is mainly expressed in the gastric tissue of human body, by analyzing the data of The Human Protein Atlas (Supplementary Figure 1a). By immunofluorescent labeling of ASAP3 and proton pump, a marker of gastric parietal cells, we confirmed that ASAP3 is specifically expressed in parietal cells (Supplementary Figure 1b). We generated the mutant allele of ASAP3 by replacing the exon 5 (starting from codon 172) with loxP-flanked sequence, and used homologous recombination in murine embryonic stem cells to create ASAP3^loxP/loxP^ model in a C57BL/6 genetic background (Figures 1b and c). Transgenic mice expressing UBC-ERT-CRE were then crossed with ASAP3 ^loxP/loxP^ mice to generate inducible knockout model (Figure 1d). The CRE recombination upon tamoxifen results in the disruption of partial BAR domain (including dimerization residues) and other regions including the PH, ArfGAP and ANK domains. A recent in-depth study has extensively characterized the BAR domain of ASAP3/ACAP4 in great details.^21^ Since the function of BAR domain is dependent on its homo-dimerization and the presence of a PH domain, the disruption ensured that the mutant allele would be functionally null. As described previously, ASAP3 expression is mainly detected in gastric parietal cells and testis leydig cells.^22,23^ We found that ASAP3^−/−^ mice were viable and fertile, without obvious morphological abnormalities. We induced ASAP3 knockout in mice by periodical injection of tamoxifen, and confirmed efficient knockout of ASAP3 in gastric tissue (Figure 1e and Supplementary Figure 2).

### Elongated actin structure and hyperactive Arf6 in resting parietal cells

**Figure 2:**
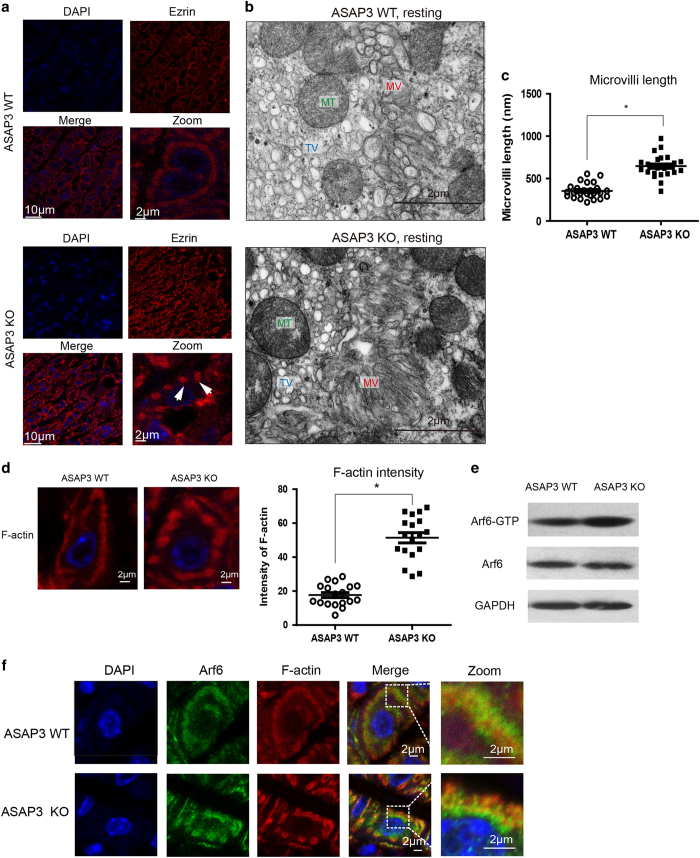
Abnormal distribution of ezrin, elongated actin structure and hyperactive Arf6 in resting ASAP3-deficient cells. (**a**) Immunofluorescence using specific antibodies for ezrin (red) in gastric parietal cells of ASAP3-deficient mice and control wild-type (WT) mice. Note the staining of ezrin displayed sac-like pattern, with the center occupied by a negatively stained region (white arrows). Scale bars are labeled within immunofluorescent images. (**b**) Transmission electron microscopy (TEM) analysis of resting parietal cells in WT mice (top) and ASAP3-deficient mice (bottom). While WT parietal cells showed short microvilli structure (MV), ASAP3-deficient parietal cells exhibited broader regions that were occupied by elongated and tightly stacked microvilli. MT, mitochondria; TV, tubulovesicle. Scale bars indicate 2 μm within TEM images. (**c**) Statistical analysis of longitudinal microvilli lengths (*n*=25 per genotype) in resting parietal cells measured by ImageJ in WT and ASAP3-deficient mice as determined by TEM (*P<0.01, Student’s *t*-test). (**d**) Representative immunofluorescent image of F-actinin resting parietal cells (left) and statistical analysis of F-actin intensity (right). Note the discontinuous staining pattern of F-actin (red) in ASAP3-defecient mice but not WT mice. Statistical analysis of F-actin intensity (*n*=18 per genotype) in multiple resting parietal cells based on immunostaining in ASAP3-defecient and WT mice (*P<0.01, Student’s *t*-test). (**e**) The expression levels of active Arf6-GTP-bound pattern, pan Arf6 in ASAP3-defecient and WT mice without stimulation were detected by Western Blot. The GAPDH level was used as loading control. The data are representative of immunoblots from nine littermates per loading group. (**f**) Representative immunofluorescent image of Arf6 (green) and F-actin (red) in resting parietal cells. Cell nucleus was stained by 4′,6-diamidino-2-phenylindole (DAPI) in blue. Zoomed sections are shown on the right panel. Scale bars indicate 2 μm in all panels.

Based on the ezrin-ASAP3 interaction indicated by the former *in vitro* study of isolated rabbit parietal cells, we first checked the structure of ezrin which prominently residues closely to apical membrane in ASAP3-deficient parietal cells at resting status. While ezrin exhibited sequential location in wild-type (WT) cells, the ASAP3-deficient parietal cells displayed its overall fragmented and concentrated distribution, with visible sac-like pattern in magnified sections, which suggested abnormalities in acid-secreting membrane networks (Figure 2a). To investigate these intriguing changes in depth, transmission electron microscopy (TEM) study was engaged to identify the subcellular equipments of the acid secretion system in parietal cells. Surprisingly, clusters of elongated microvilli were tightly crowded in ASAP3-deficient parietal cells, contrast to the normal microvilli in line inside WT parietal cells (Figure 2b). The quantification of the average microvilli length also revealed the prolongation of the microvilli overall (Figure 2c). Since F-actin is known to be the major component of cytoskeletons sustaining the apical membrane, we questioned whether actins were rearranged in elongated microvilli. Indeed, sole staining of F-actin showed a more discontinuous and concentrated staining in ASAP3-defecient mice, and statistical analysis indicated grossly enhanced intensity of F-actin compared to WT mice (Figure 2d), which demonstrated the existence of actin rearrangement in relation to microvilli distortion. Meanwhile, co-staining of F-actin and H^+^-K^+^-ATPase exhibited slightly exclusive distribution as compared to their partial colocalization in WT mice (Supplementary Figure 2). Of note, H^+^-K^+^-ATPase is packaged primarily on tubulovesicular membranes in unstimulated phase,^24,25^ thus the expanded region of F-actin and ezrin cystic-like pattern that lack H^+^-K^+^-ATPase staining should be occupied by stacks of dense microvilli visualized in TEM.

Since Arf6 GTPase (a substrate of ASAP3) has been found to regulate actin remodeling,^26–28^ we speculated that ASAP3 knockout may lead to Arf6 overactivation and thus promote actin structures. Hence we tested whether ASAP3 deletion resulted in Arf6 hyperactivation in these cells. The activated Arf6-GTP-bound pattern turned out to be significantly increased in ASAP3-defecient mice, while the total Arf6 expression remained still (Figure 2e). On the other hand, Arf6 has been suggested to colocalize with tubulovesicles that carry H^+^-K^+^-ATPase,^12^ thus we further examined Arf6 localization. In ASAP3-defecient parietal cells compared to WT cells, the immunofluorescent staining of total Arf6 displayed in a more diffuse pattern with increased portion absent from the F-actin labeled apical membrane, which was similar to that change present in H^+^-K^+^-ATPase distribution (Figure 2f, Supplementary Figures 2 and 3). Taken together, these findings supported a scenario wherein ASAP3 disruption-induced hyperactivation of Arf6 and therefore may induced F-actin rearrangement, elongation of microvilli and more diffuse H^+^-K^+^-ATPase distribution.

### ASAP3 disruption resulted in impaired canaliculi morphology and H^+^-K^+^-ATPase translocation in stimulated phase

**Figure 3:**
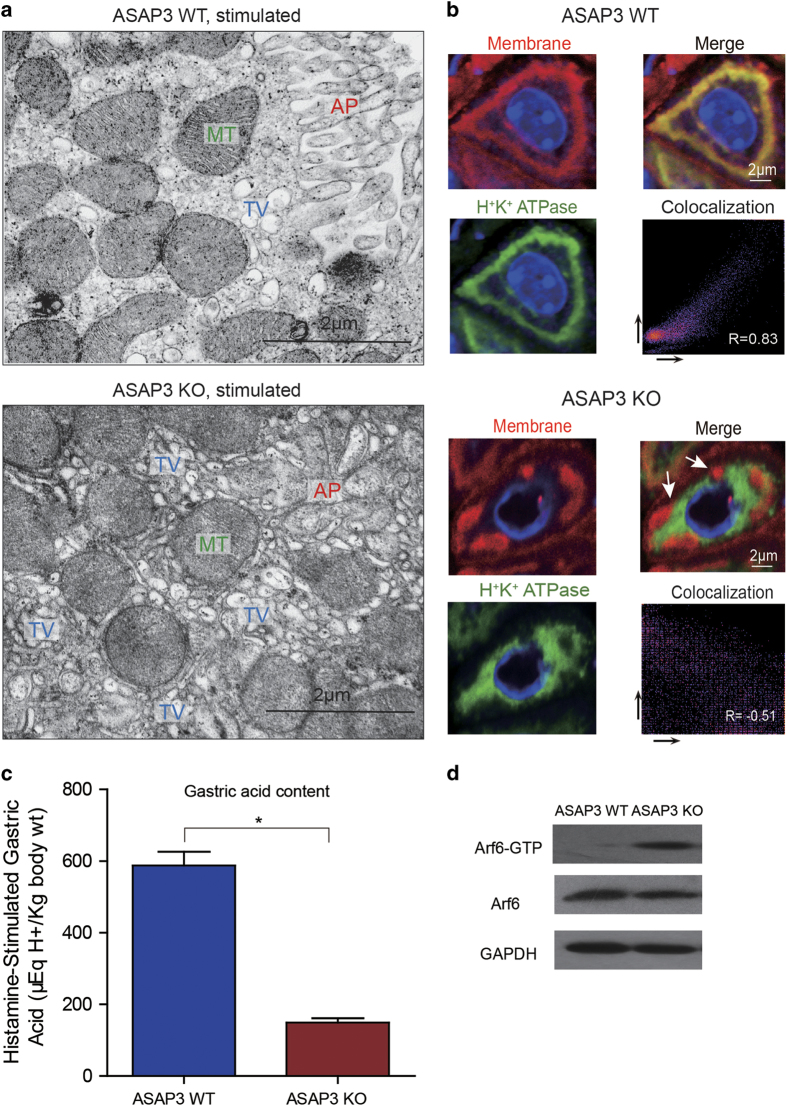
Impaired tubulovesicle fusion and H^+^,K^+^-ATPase translocation in ASAP3-deficient parietal cells upon histamine stimulation. (**a**) Transmission electron microscopy (TEM) analysis on the structures of secretory membrane in histamine-stimulated parietal cells of wild-type (WT) mice (top) and ASAP3-deficient (bottom) mice. Note the impaired tubulovesicle (TV) fusion to the abnormal apical membrane (AP) and collapsed intracellular luminal space upon stimulation in ASAP3-deficient parietal cells. In contrast, the intracellular tubulovesicle proportion decreased and normal secretory canaliculi lined up in stimulated WT parietal cells compared to its resting state. MT, mitochondria. Scale bars are indicated in the TEM images. (**b**) Immunofluorescent image and colocalization analysis of apical membrane and H^+^,K^+^-ATPase in histamine-stimulated parietal cells of WT mice (top) and ASAP3-deficient (bottom) mice. The apical membrane is labeled with a specific antibody for calcium pump pan PMCA ATPase (red), and H^+^,K^+^-ATPase is stained in green. Cell nucleus was stained by 4′,6-diamidino-2-phenylindole (DAPI) in blue. Note the mutual exclusive distribution of apical membrane and H^+^,K^+^-ATPase in stimulated ASAP3-deficient parietal cells. White arrows indicate apical membrane structures that are negatively stained for H^+^,K^+^-ATPase. The *R* values of Pearson’s correlation coefficients for red versus green panels are indicated. (**c**) Decreased gastric acid content from the stomachs of ASAP3-deficient mice in contrast to WT mice as control. Values (mean±s.e.m.) were normalized to body weight. Mice were treated with histamine (*n*=9 per genotype) to measure stimulated acid contents, respectively (*P<0.01, Student’s *t*-test). (**d**) The expression levels of active Arf6-GTP-bound pattern, pan Arf6 in ASAP3-defecient and WT mice upon histamine stimulation were detected by western blot. The GAPDH level was used as loading control. The data are representative of immunoblots from nine littermates per group.

As the function of ASAP3 has been largely examined in response to histamine stimulation in isolated parietal cells, we further applied TEM on stimulated parietal cells of ASAP3-deficient mice and wild-type controls. Shortly after histamine stimulation, the apical membrane of parietal cells in WT mice formed numerous canaliculi, with substantially extended intracellular luminal section as expected (Figure 3a). However, the apical membrane of ASAP3-deficient parietal cells displayed dramatically swollen canaliculi structure, causing a decrease in the surface area of secretory membrane and shrinkage or vanishment of the luminal space (Figure 3a, Supplementary Figures 4 and 5). More impressively, mutual exclusion of H^+^–K^+^-ATPase and secretory membrane as marked by a mouse monoclonal to calcium pump pan PMCA ATPase was found in stimulated ASAP3-deficient parietal cells, which was in contrast to high degree of their overlap in WT cells (Figure 3b). In respect to the basic molecule networks of canaliculi including ezrin and F-actin, we applied co-staining of both proteins with H^+^–K^+^-ATPase, respectively. Consistently, H^+^–K^+^-ATPase localization remained a diffused pattern in the cytoplasm (notably absent from F-actin/ezrin-occupied region), suggesting its translocation to the apical membrane should be impaired. As a control, in WT parietal cells H^+^–K^+^-ATPase strongly overlapped F-actin/ezrin-occupied region, without apparent formation of cystic structure (Supplementary Figures 1 and 6). In brief, these data suggested ASAP3 deletion disrupted canaliculi both in shape and function, which ultimately have an impact on stimulated acid secretion. In fact, on the whole organ level, stimulated gastric acid content was significantly diminished in ASAP3-defecient mice (Figure 3c).

The Arf6-associated vesicles have been implied in transporting H^+^–K^+^-ATPase,^12^ and the inactivation of Arf6 is required for vesicle decoating and membrane fusion.^29,30^ We found that Arf6-GTP-bound pattern increased greatly in spite of the constant pan Arf6 level (Figure 3d), and Arf6 distributed mostly in the cytoplasm (adjacent to the F-actin labeled apical membrane; Supplementary Figure 3b), which together supported the notion that ASAP3 deficiency led to Arf6 hyperactivation and thereby block tubulovesicles fusion. The increased Arf6-GTP-bound pattern probably also serves as the cause for abnormal actin remodeling upon stimulation, subsequently provided structural basis for the dramatically expanded canaliculi structure presented in TEM.

The ASAP3 protein includes BAR-PH domains that are implied in membrane binding and curvature,^31^ but it is unknown if ASAP3 may play such a role in parietal cells. We applied molecular dynamic simulation to study the interaction between ASAP3 BAR-PH domains (structure obtained by homology modeling) and lipid bilayer, and found ASAP3 could induce the curvature of lipid bilayer with a radius of ~40 nm (Supplementary Figure 7), being comparable to previously reported ASAP1-lipid interaction.^17^ We examined the morphology of microvilli and canaliculi in stimulated WT parietal cells, and found the membrane curvature in the root and branches of microvilli (positions where ASAP3 is supposed to bind and induce cargo delivery) were also with the radius of ~40 nm (Supplementary Figure 7). In contrast, the ASAP3-deficient parietal cells displayed no membrane curvature with such specific feature. These findings suggest that ASAP3 may induce the curvature of apical membrane and coordinate vesicle fusion to adjacent membrane, coordinately providing a spacial foundation for its role in apical membrane remodeling.

### Absence of visible bulk endocytosis recycling of H^+^–K^+^-ATPase in restoring ASAP3-deficient parietal cells

Bulk endocytosis pathway to endosomes and lysosomes occurs during elevated secretory activity when clathrin-mediated endocytosis is obstructed or unable to fully compensate the large increase in membrane surface for essential H^+^–K^+^-ATPase recycling in restoring parietal cells. To evaluate the expanded canaliculi structure attributed to hyperactivated canaliculi expansion or membrane retrieval obstruction, we next cooperated dual staining of H^+^–K^+^-ATPase and a series of organelle membrane markers, respectively. We presumed that if routine membrane retrieval is hindered due to ASAP3 disruption, part of H^+^–K^+^-ATPase containing internalized bulk inclusions would be transported to endosomes, lysosomes for recycling instead, thereby may lead to increased H^+^-K^+^-ATPase and its precursor protein synthesis at endoplasmic reticulum. However, the dual staining of ASAP3-deficient parietal cells showed no significant overlap between H^+^–K^+^-ATPase and calnexin-labeled endoplasmic reticulum membrane (Supplementary Figure 8). Meanwhile, we did not observe any colocalization of H^+^–K^+^-ATPase and EEA1, which was used as an early endosome membrane marker (Supplementary Figure 8). This was also the case in H^+^–K^+^-ATPase and Lamp1 co-staining, which labeled late endosome and lysosome (Supplementary Figure 8). Correlative statistical analysis of multiple image sections revealed no significant difference between ASAP3-defecient and WT mice (Supplementary Figure 8). To note, these outcomes confirmed ASAP3 played a role in H^+^–K^+^-ATPase enriched tubulovesicles fusion to apical membrane and instead of H^+^–K^+^-ATPase recycling.

### Gross transformation of gastric mucosa in ASAP3-deficient mice

**Figure 4:**
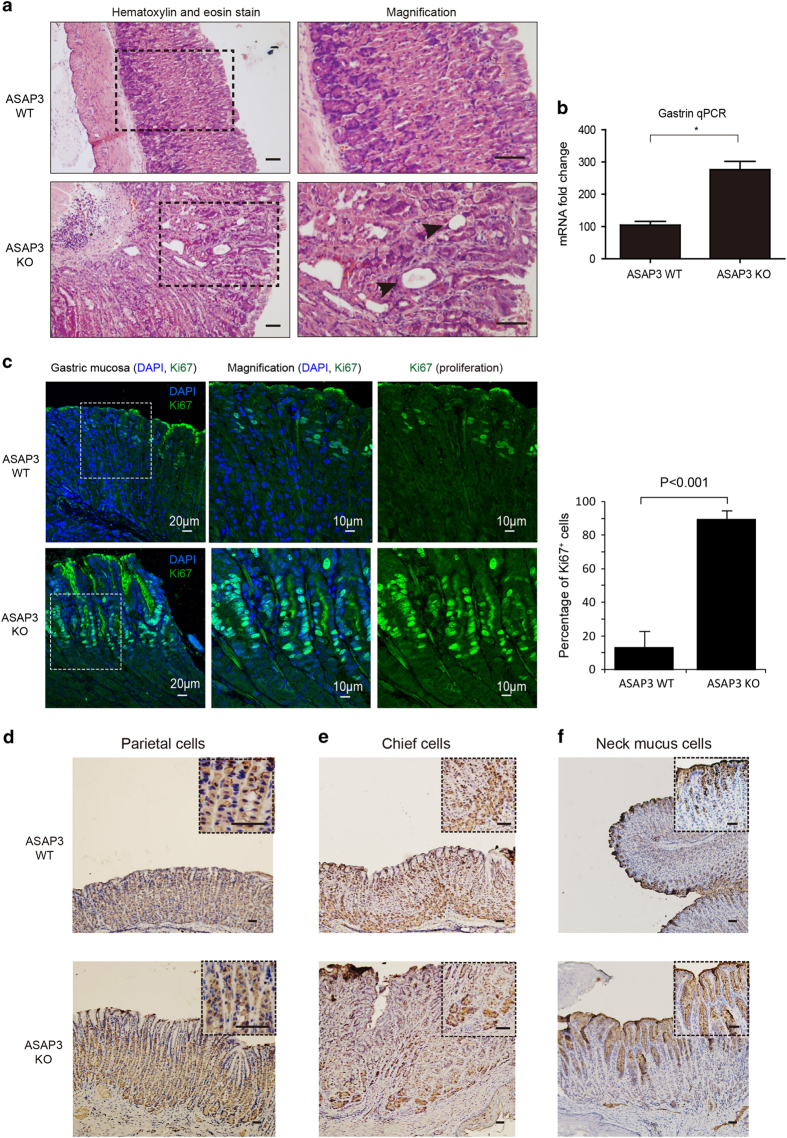
Gross histological changes in gastric mucosa of ASAP3-deficient mice. (**a**) Hematoxylin and eosin stain of gastric mucosa tissue sections from ASAP3-deficient and wild-type (WT) mice. The abnormally twisted and enlarged structures in some of the glands are marked by black arrows. (**b**) Gastrin transcript abundance was determined by quantitative reverse transcription-PCR (qRT-PCR) analysis of gastric antral RNA samples from ASAP3-deficient and WT mice (*n*=9 per genotype). Data (mean±s.e.m.) were normalized to 18S expression in the same samples and reported as fold change relative to WT. (*P<0.01, Student’s *t*-test). (**c**) Immunostaining of the Ki67 factor (green) in the gastric mucosa of ASAP3-deficient mice (bottom) and WT mice (top), the upper segment of which are adjacent to the autofluorescence of mucus in the gland duct. Comparison of the percentage of Ki67-positive cells in ASAP3-deficient mice and WT mice are shown on the right panels (*P*<0.001, Student’s *t*-test). (**d**) Sections stained for parietal cells with a monoclonal antibody (mAb) to the α subunit of H^+^,K^+^-ATPase. Insets show higher-magnification images of boxed regions. Note the abnormal distribution of H^+^,K^+^-ATPase in ASAP3-deficient parietal cells. Scale bars indicate 20 μm. (**e**) Chief cells were identified by immunostaining paraffin sections with a polyclonal antibody to intrinsic factor. Insets show higher-magnification images of boxed regions. Scale bars indicate 20 μm. (**f**) Paraffin sections were stained with Mucin5AC antibody to identify mucous cells. Insets show higher-magnification images of boxed regions. Scale bars indicate 20 μm.

Reduced gastric acidity could lead to a number of secondary changes including hypergastrinemia and gastric mucosa hyperplasia. Indeed, histological study of gastric mucosa samples revealed substantial changes in ASAP3-deficient mice compared with WT mice. Gastric hypertrophy was also found in ASAP3-deficient mice, (Figure 4a and Supplementary Figure 9). In addition, the ASAP3-deficient mucosa exhibited widespread infiltration of inflammatory cells (Supplementary Figure 10). While parietal cells were densely populated in the gland of WT mice, they were sparsely distributed in the gland of mutant mice (Figure 4a). Some of the gland in ASAP3-deletion mice appeared abnormally twisted and enlarged (Figure 4a). This could at least partially account for the loss of acidity.

Moreover, reverse transcription-quantitative PCR assays revealed significantly higher level of gastrin in mutant mice than WT controls (Figure 4b), suggesting a long-term reduction of luminal stomach acid and consequently hypergastrinemia. The thickening of gastric mucosa in ASAP3-deficient mice might be associated with hypergastrinemia.

Immunostaining of the Ki67 factor revealed active proliferation of mucous neck cells in the upper part of glands (Figure 4c). To define the chronic histopathological transformations associated with hypochlorhydria, we further analyzed the effect in terms of cell lineage changes. Comparison of parietal cell mass with H^+^–K^+^-ATPase staining showed slightly decreased parietal cell proportion in ASAP3-deficient mice (Figure 4d). In magnified sections of different populations along the ASAP3-deficient gastric glands, monoclonal antibody of H^+^–K^+^-ATPase localized its abnormal ‘vacuolar’ staining in contrast to WT (Figure 4d). It also appeared that chief cells were modestly diminished by intrinsic factor staining evaluation (Figure 4e). Consistent with increased ki67 staining, Mucin5AC staining defined the remarkably increased proportion of mucous cells in gastric glands (Figure 4f). This supported mucous cells in the surface and neck of glands to account for the thickening of gastric mucosa in ASAP3-deficient mice.

### An ArfGAP inhibitor QS11 attenuated gastric acid secretion in WT mice

**Figure 5:**
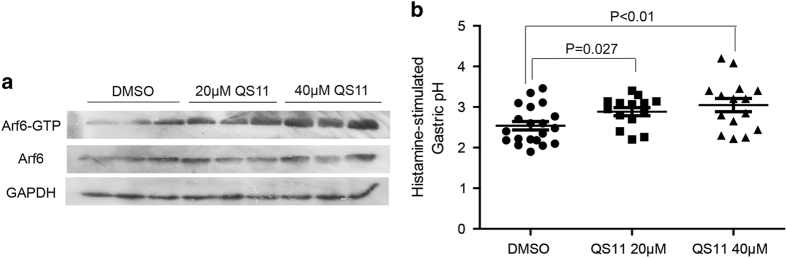
Attenuated gastric acid secretion in QS11-treated mice. (**a**) Wild-type (WT) mice were divided into three groups and consistently subjected to 20 μm QS11, 40 μm QS11 or dimethyl sulfoxide (DMSO) as control for 7 days. After 30 min of histamine stimulation, the mice were killed and gastric tissues from three independent individuals in each group were evaluated for the expression of total Arf6, GTP-Arf6. The GAPDH blotting served as loading control. (**b**) Following intraperitoneal injection of 20 μm QS11, 40 μm QS11 or DMSO for 7 days, the histamine-stimulated gastric fluid pH in the three groups was measured respectively. QS11 elicited dose-dependent effect on the inhibition of histamine-stimulated gastric acid secretion as compared to DMSO as control (*P*-value indicated in the figure, Student’s *t*-test). The uneven effects of QS11 administration might be due to the variable levels of total Arf6 in different individuals.

To further validate the potential significance of ASAP3 in gastric acidity control, we employed a small-molecule ArfGAP inhibitor QS11 to mimic the ablation of ASAP3 *in vivo*. The incremental doses of QS11 were given to respective groups of mice intraperitoneally at 20 μm or 40 μm for 7 days. Although the effect of QS11 at 20 μm was modest (*P*=0.027, Student’s *t*-test), 40 μm QS11 effectively evoked a decrease in the histamine- stimulated gastric acid secretion (Figure 5a). Although QS11 was not specific for sole Arf6, analysis of Arf6 activation status revealed a dose-dependent increase of GTP-bound Arf6 level in QS11-treated mice, albeit with unchanged total Arf6 expression (Figure 5b). In concordance with the elevated Arf6-GTP levels in achlorhydric ASAP3-defecient mouse model, the diminished gastric acid content in QS11-treated mice strongly recommended an essential role of the transformation between Arf6-GTP and Arf6-GDP in maintenance of gastric fluid acidity.

### ASAP3 is upregulated in patients with reflux or gastric ulcer

**Figure 6:**
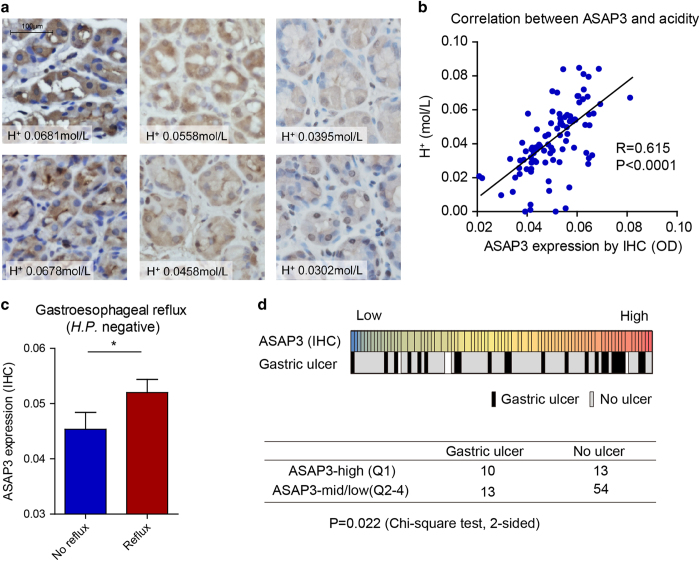
Clinical relevance of ASAP3 in gastric acidity. (**a**) Representative immunohistochemistry (IHC) staining images for ASAP3 in gastric mucosa tissue, with quantified acid content indicated on the bottom of each image. The scale bar representing 100 μm applies to all images. (**b**) Correlation between ASAP3 expression and basal gastric acid secretion. Insets show higher-magnification images of boxed regions. The expression of ASAP3 expression was determined by IHC of tissue specimens (*n*=90), and the gastric acid concentration of overnight-fasting patients was determined by titration. The *R* value for Pearson correlation analysis is indicated in the plot (*P*<0.0001). (**c**) The expression of ASAP3 determined by IHC was compared between Helicobacter pylori-negative cases wither with or without gastro-esophageal reflux disease (GERD)/reflux (*P<0.05, two-sided *t*-test). (**d**) High expression of ASAP3 (top 25%, Q1) associated with gastric ulcer in human. The heat map in upper panel shows the expression of ASAP3 and gastric ulcer in patients, and the lower panel shows the result of chi-square test.

To determine whether ASAP3 may affect gastric acid secretion in human, we measured ASAP3 expression in gastric endoscopic tissue specimens by immunohistochemistry and analyzed its association with gastric acid secretion. To this end, the gastric fluids of overnight-fasting patients were sampled through endoscopy and measured for pH and total acid content by titration. Of note, the expression level of ASAP3 strongly correlated with gastric acid content (Pearson *R*=0.615, *P*<0.0001, Figures 6a and b). As *Helicobacter* *Pylori* may be related to GERD, we compared ASAP3 expression in GERD and non-GERD cases who had no history of *H. Pylori* infection. Interestingly, the expression of ASAP3 was significantly higher in GERD patients than in non-GERD cases (Figure 6c). The subjects expressing high level of ASAP3 (top 25%) were found with significantly more gastric ulcer than other subjects (Figure 6d). These results collectively demonstrate that ASAP3 associates with gastric acidity in human, and its deregulation may associate with GERD and gastric ulcer.

## DISCUSSION

By combined *in vivo* investigation approaches, we provide the first evidence for ASAP3’s role in regulating the microvilli and apical membrane structures. These results also revealed ASAP3 as a feasible and drugable intervention target for controlling gastric acidity. Both targeted disruption of *ASAP3* gene in mice and pharmaceutical inhibition of ASAP3 by QS11 small molecular compound demonstrated consistent effect on the suppression of gastric acidity. Moreover, the strong correlation between ASAP3 expression and human gastric acid secretion implicated ASAP3 as a potential therapeutic target in clinically refractory acid-related diseases.

**Figure 7:**
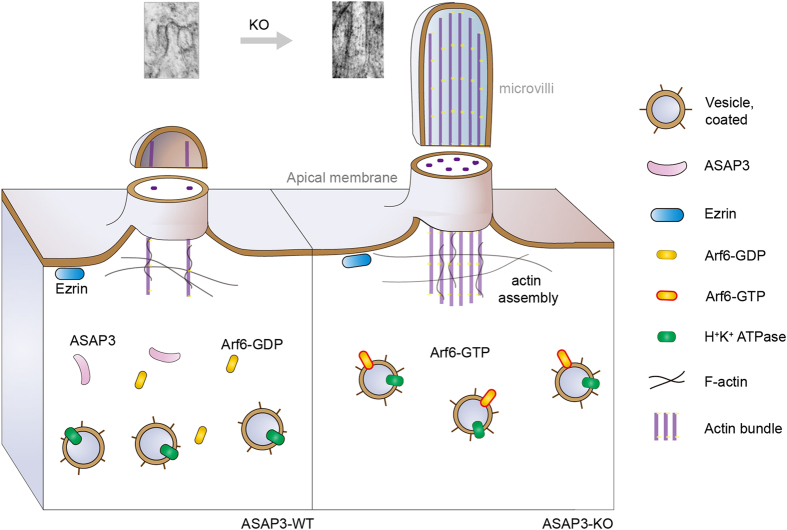
Schematic representation of ASAP3’s roles in parietal cells. The novel roles of ASAP3 in regulating microvilli and apical membrane structures in resting parietal cells. In wild-type (WT) parietal cells (left), the Arf6 GTPase cycles between GTP-bound active form and GDP-bound inactive status. Controlled assembly of actin induces the formation of short microvilli. However in parietal cells of ASAP3-deficient mice (right), disruption of ASAP3 impairs its ArfGAP function, thus causing overactivation of Arf6 (Arf6-GTP). This enhances actin polymerization and induces the formation of elongated and tightly stacked microvilli. The representative transmission electron microscopy (TEM) images in two conditions are also shown.

Our *in vivo* study provided novel insight into the multifaceted ASAP3 functions in gastric parietal cells, which are involved in the regulation of microvilli and apical membrane structures. Transmission electronic microscopy of tissue sections from ASAP^−/−^ mice revealed that microvilli were significantly elongated and stacked in resting parietal cells. The GTP-bound, activated form of Arf6 has been found to promote actin polymerization,^32^ thus ASAP3 disruption-induced Arf6 activation may contribute to the elongation of actin and microvilli in resting parietal cells (schematic representation in Figure 7). This effect of ASAP3 was not reported in the previous study,^16^ potentially due to the *in vitro* isolation procedures that may disturb the ultrastructure of microvilli. In resting parietal cells, the mislocalization of H^+^–K^+^-ATPase seems to be caused by the occupational effect of the elongated and stacked microvilli structure. Although several members of the ArfGAP family have been found to modulate Arf6 activity, our data suggest that there is no redundancy of function in this instance: that neither ASAP1 nor any other ArfGAP can adequately compensate for loss of ASAP3 in parietal cells. Of note, the roles of ASAP3 in regulating apical membrane and microvilli structures have not been reported before, and our data suggest that ASAP3 plays multifaceted roles mediated by its interaction with apical membrane and the regulatory effect on Arf6 activity.

The ASAP3-deficient mice also revealed significant ultrasturctural abnormality of apical membrane in stimulated parietal cells. We observed increased tubulovesicles in the cytoplasm of stimulated ASAP3^−/−^ mice, therefore confirmed the previous observation on the requirement of ASAP3 for H^+^–K^+^-ATPase translocation.^16^ In addition, we also observed the expansion of canaliculi and the shrinkage/collapse of intracellular luminal space also contributed to the decreased gastric acid secretion. We propose that this may be due to the alteration of actin structure within the microvilli, which still deserves further investigation in future studies. Disordered apical membrane was also found in a previous ezrin^kd/kd^ mouse model.^14^ Regarding the high mortality and severe growth retardation in ezrin^kd/kd^ mice,^16^ our results suggested that ASAP3 should be a more feasible target for decreasing gastric acidity.

As mentioned previously, deregulation of gastric acid secretion may result in some of the most prevalent diseases such as GERD and peptic ulcer. Among the compounds that inhibit acid secretion, acid susceptible PPIs, such as the substituted benzimidazoles, omeprazole, lanoprazole, rabeprazole and pantoprazole are the most prominent and effective ones. However, despite their claimed efficiency, phenomena of acid rebound and tolerance are major drawbacks to the use of these drugs that has recently come into light. It has been estimated that ~30% of GERD patients remain symptomatic on standard dose of PPI,^33,34^ thereby making it important to identify alternative therapeutic targets for more robust and durable gastric acidity intervention. In our study, a small-molecule inhibitor of ASAP3 successfully decreased gastric acid secretion *in vivo* and mimicked the effect of ASAP3 knockout. Moreover, our data demonstrated the expression of ASAP3 significantly associated with the level of gastric acid secretion in human biopsies. These results collectively suggest that targeting ASAP3 by small molecular inhibitor such as QS11 may provide alternative strategy to control gastric acid-related diseases.

In summary, our data suggest novel roles of ASAP3 in gastric parietal cells, and revealed ASAP3 as a feasible and drugable target for the control of gastric acidity and related disorders.
