# Supplementary material for: ASAP3 regulates microvilli structure in parietal cells and presents intervention target for gastric acidity
Source: Signal Transduct Target Ther. 2017 Feb 24;2:17003–. doi: 10.1038/sigtrans.2017.3 (PMC5661632; doi:10.1038/sigtrans.2017.3)
Supplement: Supplementary Information [file sigtrans20173-s1.doc]

**Supplementary Information**

**ASAP3 regulates microvilli structure in parietal cells and presents intervention target for gastric acidity**

Jin Qian^1,2^, Yueyuan Li^1,2^, Han Yao^1^, Haiying Tian^1^, Huanbin Wang^1^, Luoyan Ai, Yuanhong Xie ^1^, Yujie Bao, Lunxi Liang^1^, Ye Hu^1^, Yao Zhang^1^, Jilin Wang, Chushu Li^1^, Jiayin Tang^1^, Yingxuan Chen^1^, Jie Xu^1^*, Jing-Yuan Fang^1^*

^1^ Division of Gastroenterology and Hepatology, Key Laboratory of Gastroenterology and Hepatology, Ministry of Health,State Key Laboratory for Oncogenes and Related Genes,Renji Hospital, School of Medicine, Shanghai JiaoTong University; Shanghai Institute of Digestive Disease;145 Middle Shandong Road, Shanghai 200001, China

^2^JQ and YL contributed equally to this work

Correspondence to:

Jing-Yuan Fang (jingyuanfang@sjtu.edu.cn) or Jie Xu (jiexu@sjtu.edu.cn), Renji Hospital, School of Medicine, Shanghai Jiao Tong University, Shanghai

**Supplementary Materials and methods**

**Transmission electron microscopy (TEM) analysis**

The ultrastructure of parietal cells in WT and ASAP3-deficient mice were analyzed by TEM using a method reported previously. Mice were left untreated (basal) or treated with histamine (stimulated; 20 mg/kg in PBS) by peritoneal injection 30 min prior to sacrifice. Fundic tissue strips were cut and fixed overnight at 4°C in 2% glutaraldehyde plus 2% PFA in 0.1 M sodium cacodylate buffer (pH 7.4), followed by washing in 0.1 M sodium cacodylate buffer. Tissue was treated with 4% osmium tetroxide, washed in 0.1 M sodium cacodylate buffer, briefly rinsed in deionized water, and dehydrated and stained with 2% uranyl acetate in 70% ethanol, followed by a graded ethanol series and embedding in Epon/Araladite using standard polypropylene oxide infiltration procedures. Sections (70-80 nm) were stained with uranyl acetate and lead citrate and visualized with either a JEOL 1010 or a JEOL 1230 transmission EM equipped with a Gatan 16-megapixel digital camera. At least 25 parietal cells from 2 independent tissue sections obtained from each of 4 animals were analyzed for each genotype and treatment.

**Quantification of mouse gastric acid secretion**

Quantification of mouse gastric acid secretion was determined using a method reported previously [^3^](#_ENREF_3). Mice of 7-8 weeks old were fasted for 24 h and then injected with histamine (2mg/kg). After 30 min, the gastroesophageal and pyloric junctions were clamped, and the stomach was removed. The stomach was immersed in 2 mL normal saline and opened along the lesser curvature. The solution containing the gastric contents was centrifuged at 500 × g for 15 min, and the supernatant was recovered for measuring pH and titrating to pH 7.0 with NaOH; the titration value was normalized to the stomach wet weight.

**Administration of QS11 in WT mice**

C57BL/6 genetic background mice (7-8 weeks) were randomly divided into three groups. Treatments were either vehicle (50% DMSO/saline*150μL IP QD), QS11 (abcam141408) (20μM* 150 μL IP QD) and QS11 (40μm*150 μL IP QD) for each mice. Due to the limited access to pharmacokinetic and pharmacologic data of QS11 on mammalian administration, the concentration in our study referred to a validated *in vitro* administration of QS11[^8^](#_ENREF_8). After 7 days of continuous injection, the mice were sacrificed and measured for gastric acid secretion after 30 minutes of subcutaneously histamine stimulation according to the above mentioned protocol. For western blot, gastric tissues from three individual mice in each group were taken and proteins were extracted.

**Gastrin expression analysis**

Blood was collected from mice of 7-8 weeks old by cardiac puncture into heparinized tubes, and plasma was isolated and centrifuged for 15 minutes at 1000 ×g at 2-8°C within 30 minutes of collection. Samples at -20°C until assayed. Gastrin levels were further assayed using RT-PCR analysis. RNA was isolated from 7-8 weeks-old WT and ASAP3-deficient mice gastric corpus. RT-PCR reactions were performed with PrimeScript RT reagent Kit (Takara) according to the manufacturer’s instructions, and samples were tested in triplicate. Primer sequences were as follows as previously describe[^6^](#_ENREF_6): gastrin, forward ACACAACAGCCAACTATTC, reverse CAAAGTCCATCCATCCGTAG.

**Protein homology modeling and molecular dynamic simulation**

The protein structure of ASAP3 was obtained by homology modeling using the Phyre2 algorithm [^1^](#_ENREF_1), based on the x-ray structure of ASAP1 BAR-PH domain and ASAP3 ArhGAP-ANK domain. We used a fully hydrated, equilibrated membrane phospholipid bilayer containing 256 palmitoyloleoyl PE (POPE) molecules that were described previously [^2^](#_ENREF_2).

We generated a lipid bilayer of 40% DOPC/30%DOPE/20%POPS/10% PI(4,5)P2 using a method reported previously [^5^](#_ENREF_5). MD simulations were performed using the GROMACS 4.5 package with a modified version of GROMACS force field [^4^](#_ENREF_4). Simulations were performed under constant NPT (a fixed number of atoms N, pressure P, and temperature T) conditions and periodic boundary conditions, and other simulation parameters can be found elsewhere [^7^](#_ENREF_7). Briefly, the initial velocities were taken randomly from a Maxwellian distribution at 300 K. The temperature was held constant by Berendsen coupling. A constant pressure of 1 bar was applied with a coupling constant of 1.0 ps. The Van der Waals cutoff was set to 0.8 nm, and Long-range electrostatic interactions were calculated using the particle mesh Ewald summation methods with a cut off of 1.4 nm. The pair lists were updated every 10 steps. The LINCS algorithm was used to constrain bond lengths. During energy minimization the steepest descents algorithm was used and the minima was reached in 500 steps. MD was performed with a time step of 2 fs and the coordinates were saved every 500 steps. We set the simulation time for each circle to 100 ns, and used the resultant trajectories for further analysis.

**Statistical analysis**

Data from at least three independent experiments performed in triplicate are presented as the mean standard deviation (SD). Comparisons were performed using the Student’s paired t-test. P<0.05 was considered statistically significant.

**Supplementary References**

1 Bennett-Lovsey RM, Herbert AD, Sternberg MJ, Kelley LA (2008). Exploring the extremes of sequence/structure space with ensemble fold recognition in the program Phyre. *Proteins* **70:** 611-625.

2 Murzyn K, Rog T, Pasenkiewicz-Gierula M (2005). Phosphatidylethanolamine-phosphatidylglycerol bilayer as a model of the inner bacterial membrane. *Biophysical journal* **88:** 1091-1103.

3 Nishi M, Aoyama F, Kisa F, Zhu H, Sun M, Lin P *et al* (2012). TRIM50 protein regulates vesicular trafficking for acid secretion in gastric parietal cells. *The Journal of biological chemistry* **287:** 33523-33532.

4 Pronk S, Pall S, Schulz R, Larsson P, Bjelkmar P, Apostolov R *et al* (2013). GROMACS 4.5: a high-throughput and highly parallel open source molecular simulation toolkit. *Bioinformatics* **29:** 845-854.

5 Wang JL, Chen ZF, Chen HM, Wang MY, Kong X, Wang YC *et al* (2014). Elf3 drives beta-catenin transactivation and associates with poor prognosis in colorectal cancer. *Cell death & disease* **5:** e1263.

6 Weng YR, Kong X, Yu YN, Wang YC, Hong J, Zhao SL *et al* (2014). The role of ERK2 in colorectal carcinogenesis is partly regulated by TRAPPC4. *Molecular carcinogenesis* **53 Suppl 1:** E72-84.

7 Zhou X, Xu J (2012). Free cholesterol induces higher beta-sheet content in Abeta peptide oligomers by aromatic interaction with Phe19. *PloS one* **7:** e46245.

8 Zhu W, London NR, Gibson CC, Davis CT, Tong Z, Sorensen LK *et al* (2012). Interleukin receptor activates a MYD88-ARNO-ARF6 cascade to disrupt vascular stability. *Nature* **492:** 252-255.

**Supplementary Figures**

**
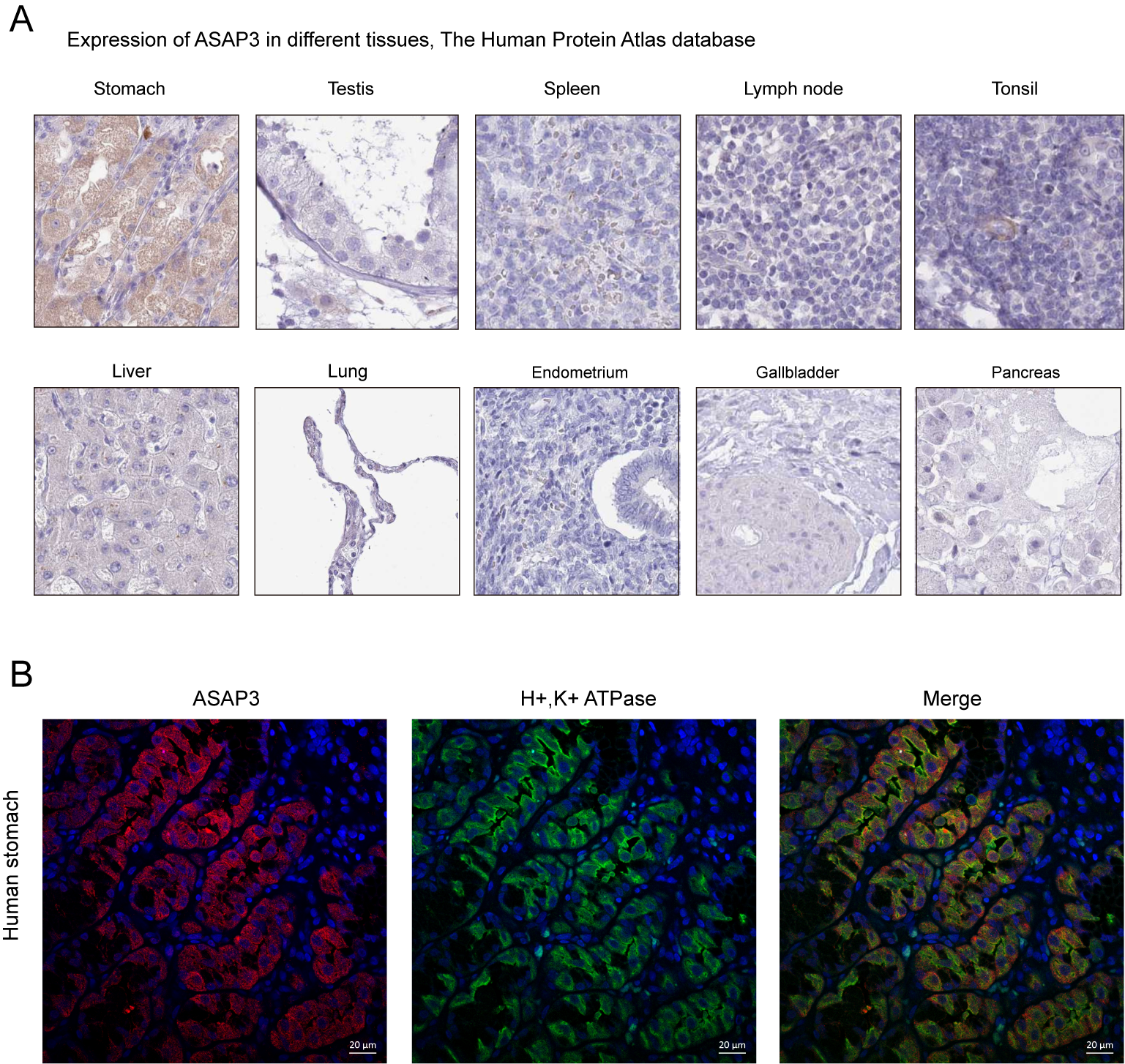
**

**Supplementary Figure 1. Expression of ASAP3 in different tissues.**

(**A**) The expression of ASAP3 in stomach, liver, testis, lung and other tissues as determined by immunohistochemistry. These representative images were obtained from The Human Protein Atlas.

(**B**) The expression of ASAP3 (labeled in red) is specifically found in parietal cells that express H^+^,K^+^-ATPase (green), as revealed by immunofluorescence.

**
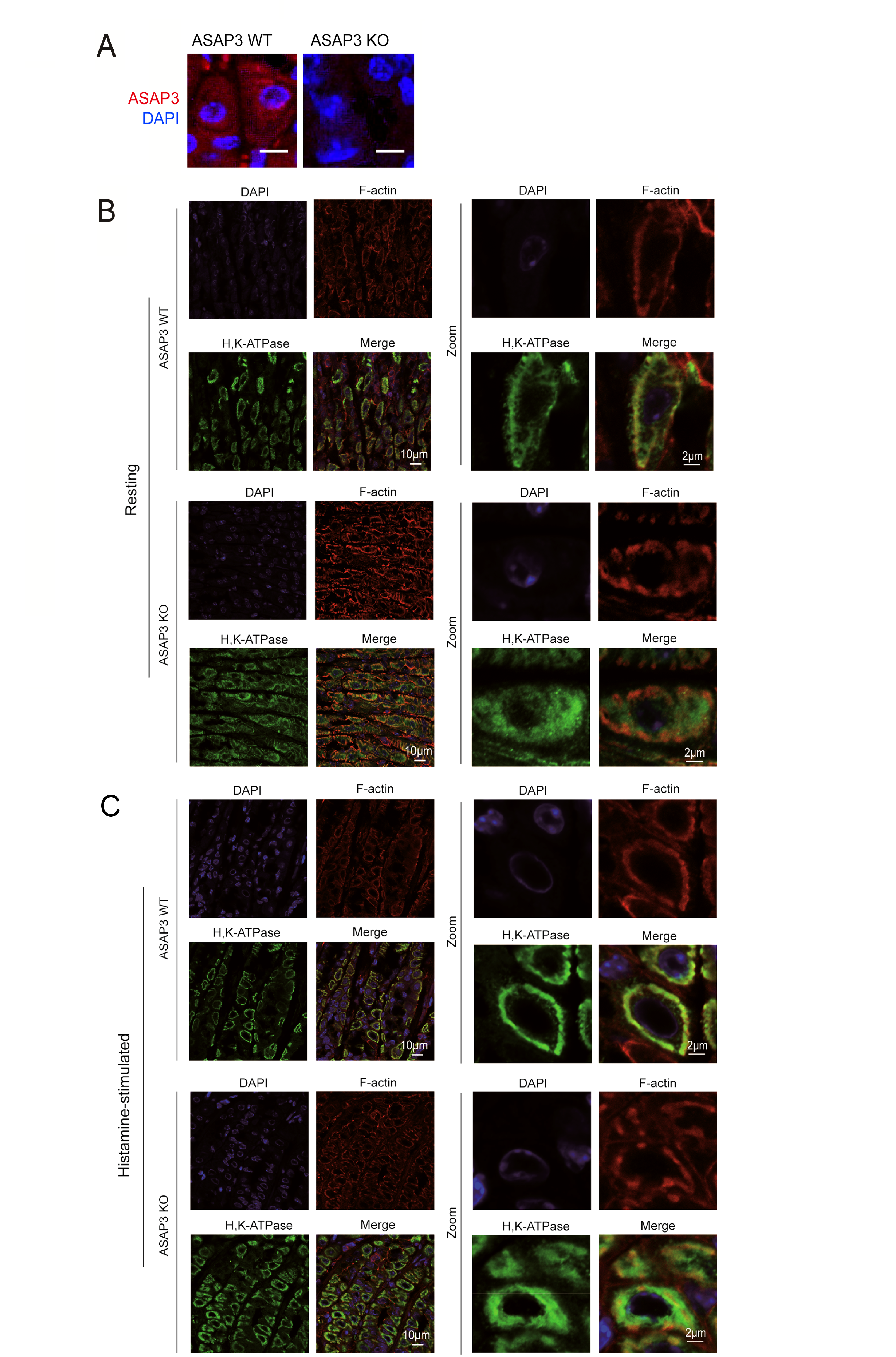
**

**Supplementary Figure 2. Abnormal intracellular canalicular formation and impaired H^+^,K^+^-ATPase translocation in ASAP3-deficient parietal cells.**

(**A**) Immunofluorescence using specific antibody for ASAP3 confirmed the knockout of ASAP3 in the gastric mucosa tissue of tamoxifen-induced mice. Cell nuclei were stained with DAPI in blue. Scale bars indicate 5µm.

(**B**) Immunostaining of H^+^,K^+^-ATPase (green) and F-actin (red) in the gastric mucosa of resting ASAP3-deficient mice (bottom) and WT mice (top). Cell nuclei were stained with DAPI in blue. Single parietal cell from these images is magnified. Note the decreased colocalization pattern in ASAP3-deficient mice compared to that in WT mice.

(**C**) Confocal immunofluorescence showing the intracellular canalicular structure in histamine-stimulated ASAP3-deficient (bottom) and WT (top) gastric glands, visualized by costaining for F-actin (red) and H^+^,K^+^-ATPase α-sununit. Cell nuclei were stained with DAPI in blue. Single parietal cell from these images is magnified. By contrast, the WT parietal cells inhibited strong colocalization of H^+^,K^+^-ATPase and F-actin, while in ASAP3-deficient parietal cells, H^+^,K^+^-ATPase and F-actin staining showed a nearly exclusive pattern. Note the saclike discontinuous staining pattern of F-actin in ASAP3-defecient mice but not in WT mice.


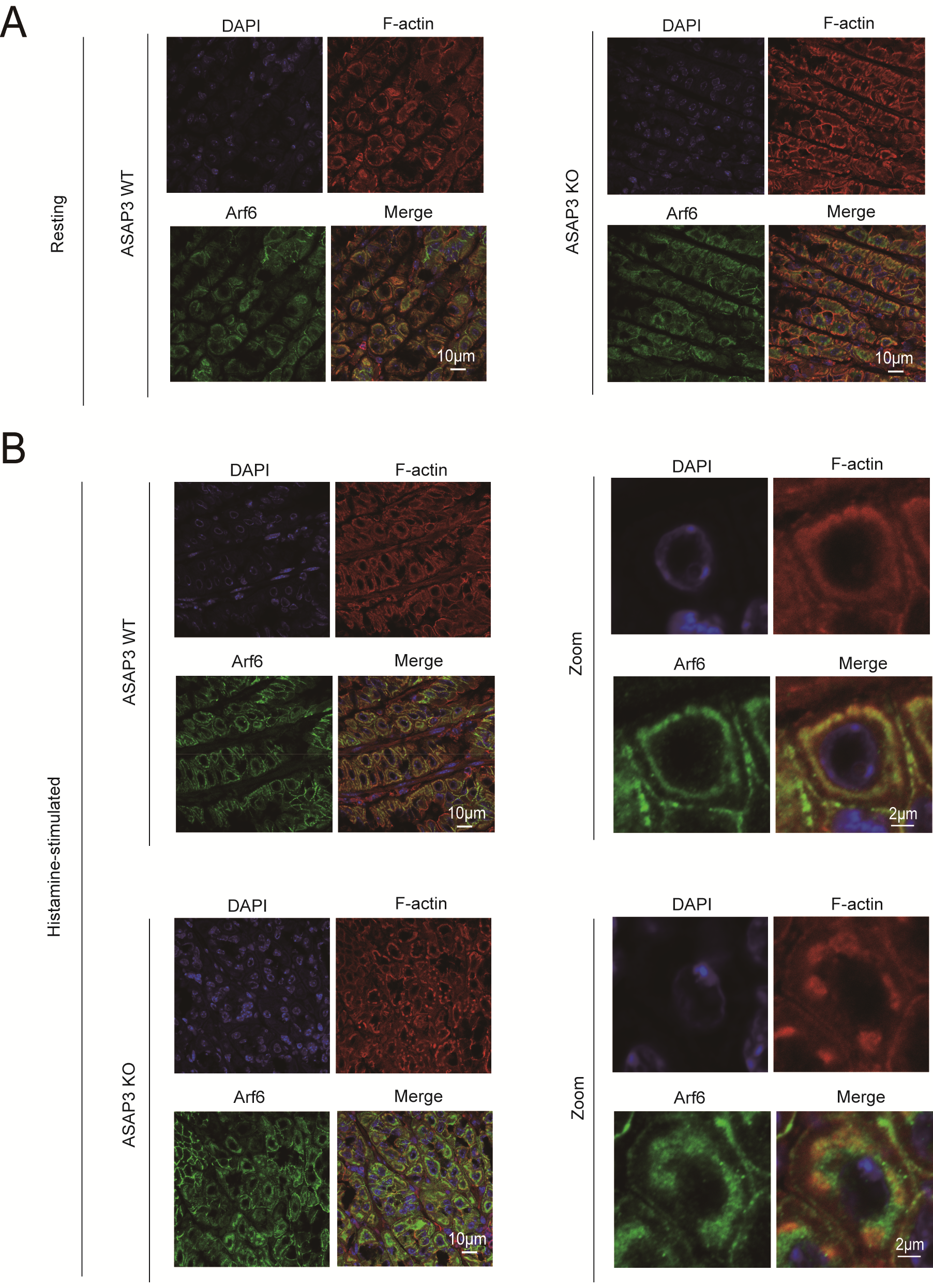


**Supplementary Figure 3. Abnormal distribution of Arf6 and F-actin in ASAP3-deficient parietal cells.**

(**A**) Confocal sections from resting ASAP3-deficient and WT mice gastric mucosa, visualized by costaining for Arf6 (green) and F-actin (red). Cell nucleus was stained by DAPI in blue. Magnification of single parietal cells obtained from these sections is shown in Figure 2G.

(**B**) Confocal sections from histamine-stimulated ASAP3-deficient and WT mice gastric mucosa, visualized by costaining for Arf6 (green) and F-actin (red). Cell nucleus was stained by DAPI in blue. Magnified images obtained from representative single parietal cells in these sections are shown in the right panel. Note the relatively diffuse distribution of Arf6 and decreased colocalization of Arf6 and F-actin in stimulated ASAP3-deficient parietal cells.


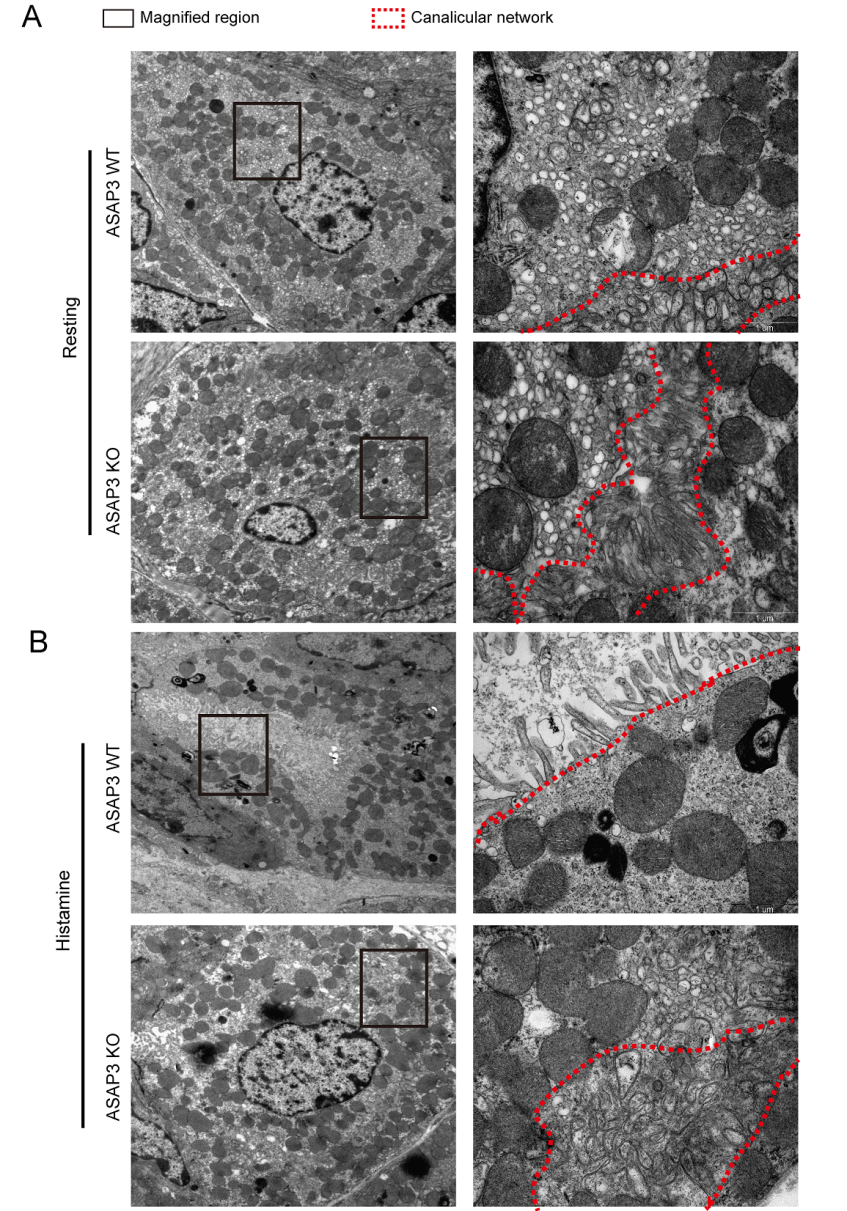


**Supplementary Figure 4. Structure of microvilli in resting and stimulated ASAP-deficient parietal cells as determined by EM**

(**A**) The morphology of canalicular network (marked in red) in resting ASAP3-deficient and wild-type parietal cells.

(**B**) The structure of apical membrane in stimulated ASAP3-deficient and wild-type parietal cells.


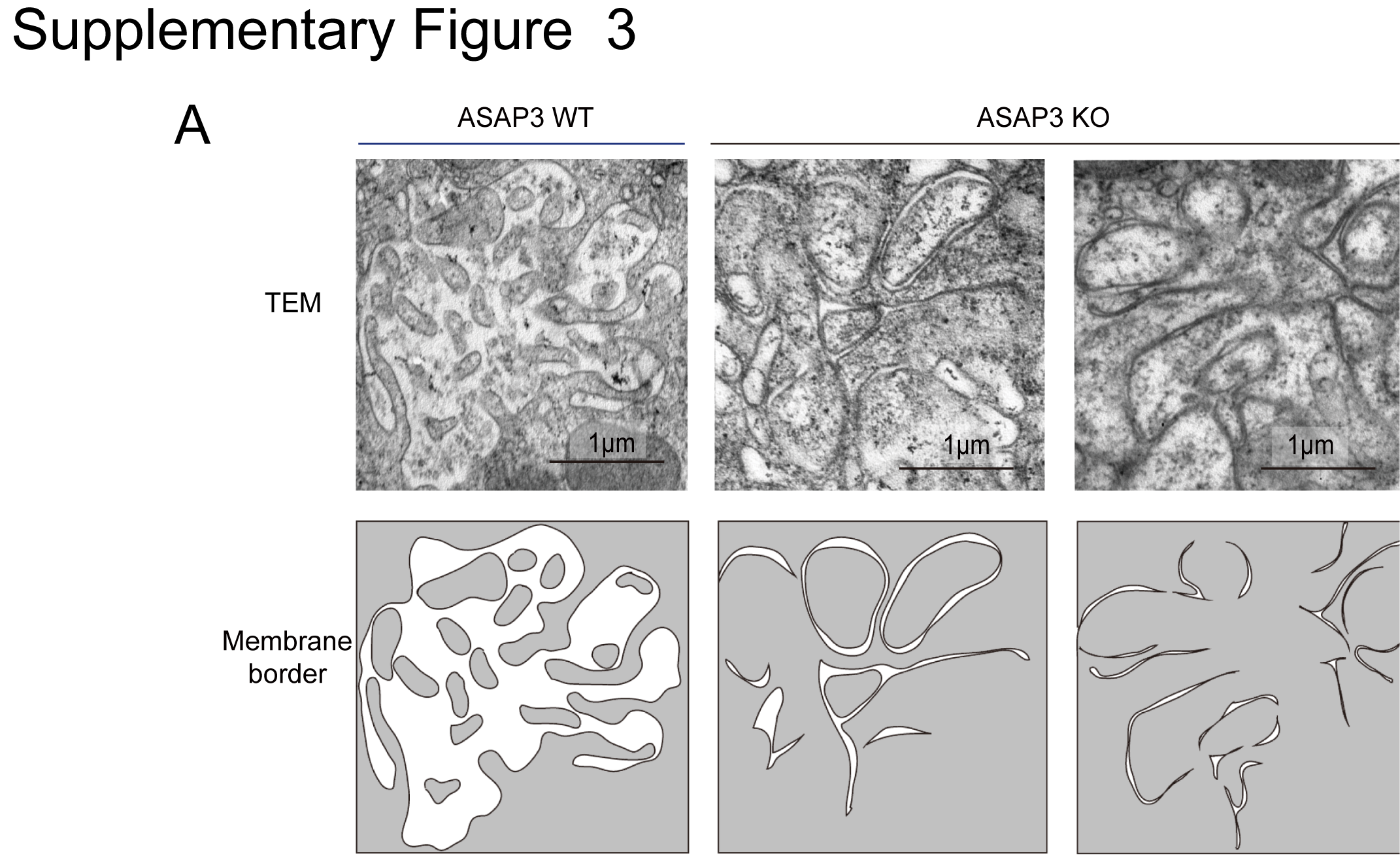


**Supplementary Figure 5. The collapse of luminal space in the secretory membrane of ASAP3-deficient parietal cells.**

TEM analysis showing the shrinkage or collapse of luminal space in the secretory membrane of ASAP3-deficient parietal cells. The membrane borders and luminal space are shown in the lower panels.


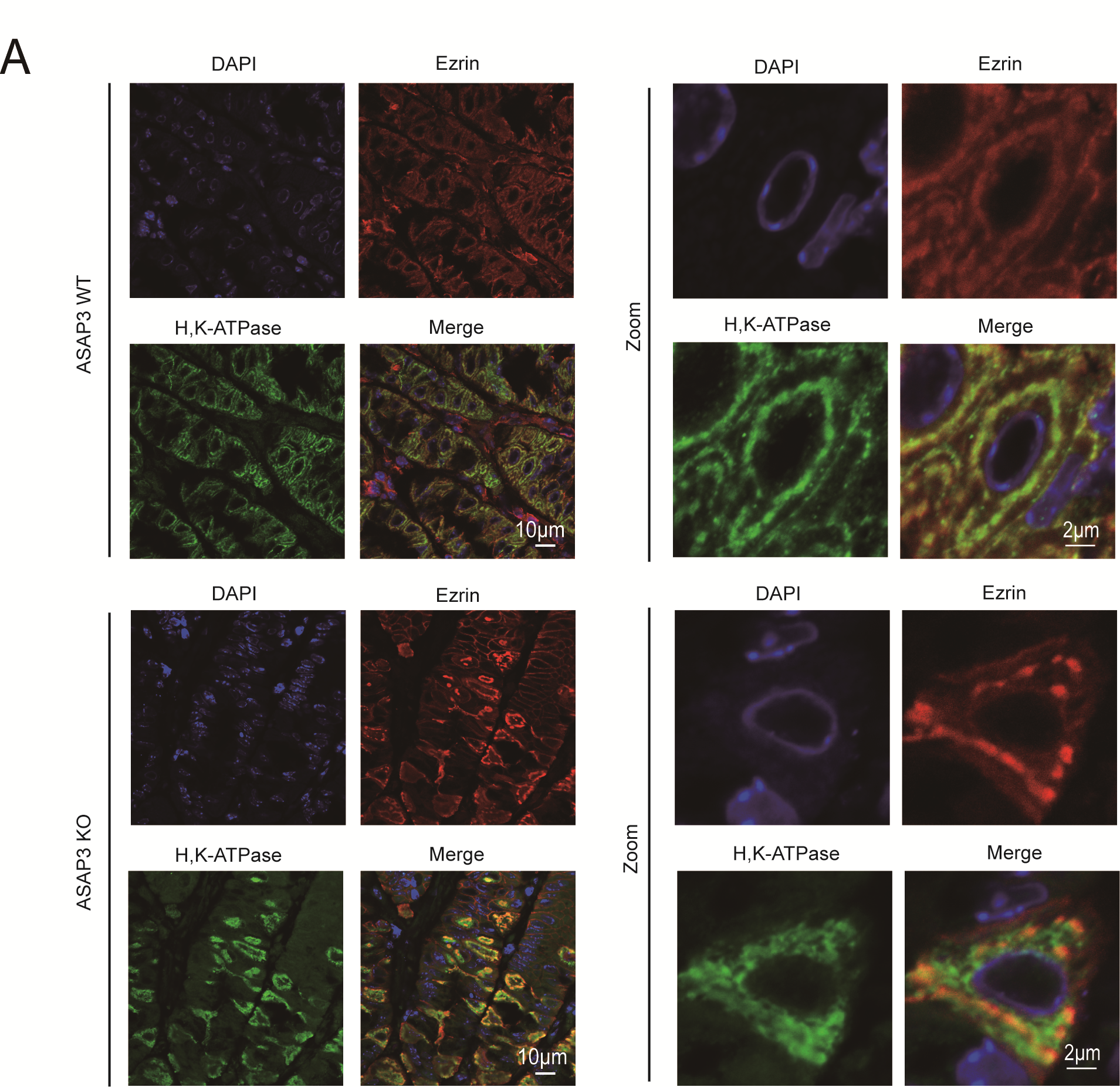


**Supplementary Figure 6. Impaired H^+^,K^+^-ATPase translocation to ezrin-labeled apical membrane in ASAP3-deficient parietal cells upon histamine stimulation.**

Representative immunofluorescent image of ezrin-labeled apical membrane (red) and H^+^,K^+^-ATPase (green) in histamine-stimulated parietal cells. The WT parietal cells inhibited strong colocalization of H^+^,K^+^-ATPase and ezrin, in contrast to the diffuse distribution of H^+^,K^+^-ATPase surrounding ezrin-labeled apical membrane in ASAP3-deficient parietal cells. Cell nuclei were stained with DAPI in blue. Magnified images obtained from representative single parietal cells in these sections are shown on the right panels.


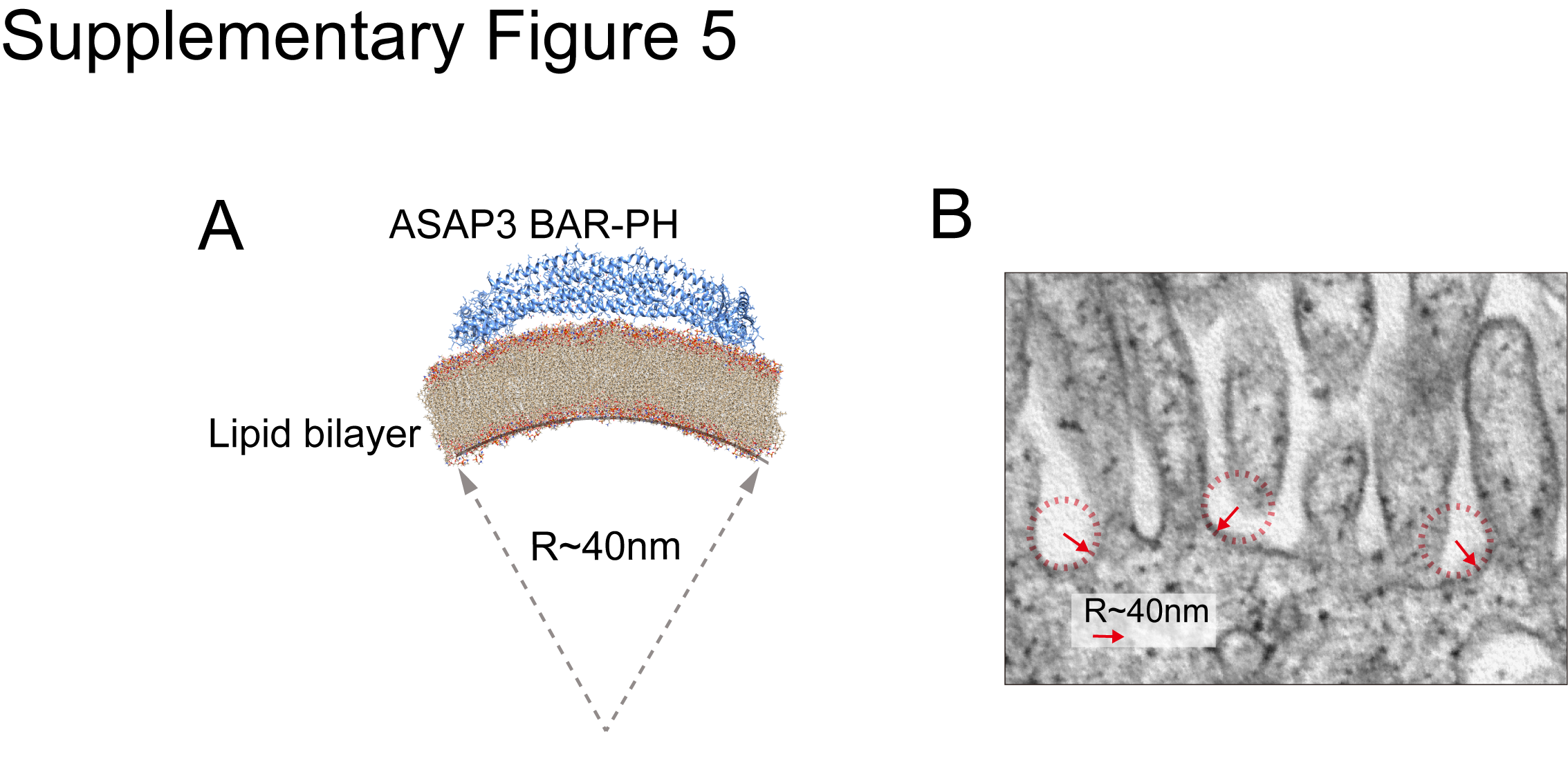


**Supplementary Figure 7. Structural analysis of ASAP3 protein domains.**

(**A**) Structure of ASAP3 homodimer and lipid bilayer complex determined by molecular dynamics. The ASAP3 BAR-PH domain was constructed by homology modeling based on ACAP1 (4nsw.pdb). After the simulation time of 100 ns, the BAR-PH domain induced curvature of the lipid bilayer with a radius close to 40nm.

(**B**) Curvature of membrane with a radius of ~40nm on one side of the root segment of microvilli (red arrows).

**
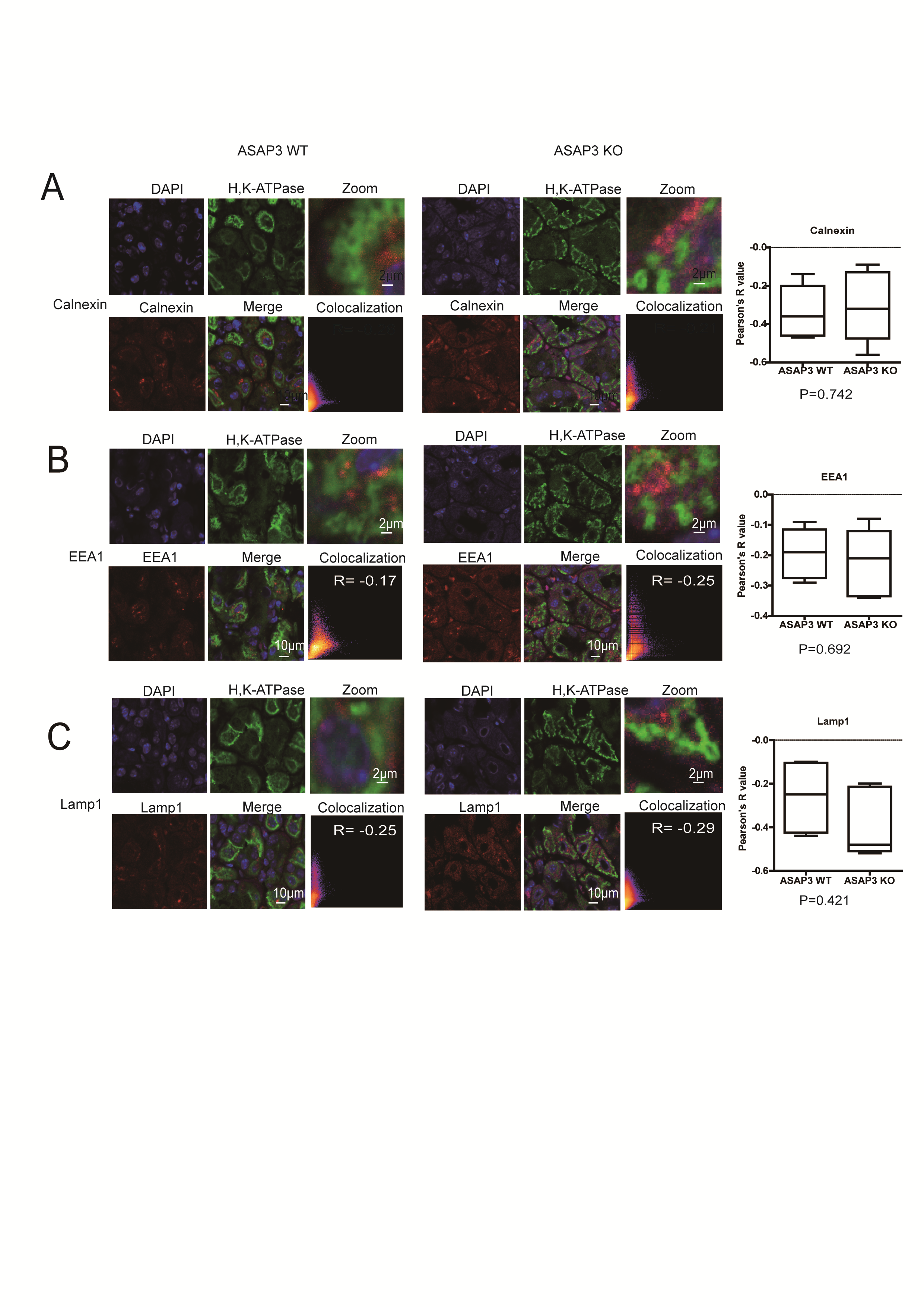
Supplementary Figure 7**. **Abnormal H^+^,K^+^-ATPase restoring was excluded in ASAP3-deficient parietal cells .**

(**A**) Immunofluorescent detection and colocalization analysis of H^+^,K^+^-ATPase (green) and calnexin(red) as an endoplasmic reticulum membrane marker in restoring ASAP3-defecient(right panel)and WT (left panel) parietal cells. The R values of Pearson's correlation coefficients for red vs. green panels are indicated. Statistical analysis of Pearson’s coefficient acquired from multiple immunostaining sections (n=5 per genotype) are shown on the right panels (P=0.742, student t-test).

(**B**) Immunofluorescent detection and colocalization analysis of H^+^,K^+^-ATPase (green) and EEA1(red) as an early endosome membrane marker in restoring ASAP3-defecient (right panel)and WT (left panel) parietal cells. The R values of Pearson's correlation for red vs. green panels are indicated. Statistical analysis of Pearson’s coefficient acquired from multiple immunostaining sections (n=5 per genotype) shows no significant difference between ASAP3-defecient and WT mice (P=0.692, student t-test).

(**C**) Colocalization analysis of H^+^,K^+^-ATPase (green) and lamp1(red) in ASAP3-defecient(right panel)and WT (left panel) gastric tissues. Lamp1 antibody was used to define endosome and lysosome membrane. The R values of Pearson's correlation for red vs. green panels are indicated. Statistical analysis of Pearson’s coefficient acquired from multiple immunostaining sections (n=5 per genotype) are shown on the right panels (P=0.421, student t-test).


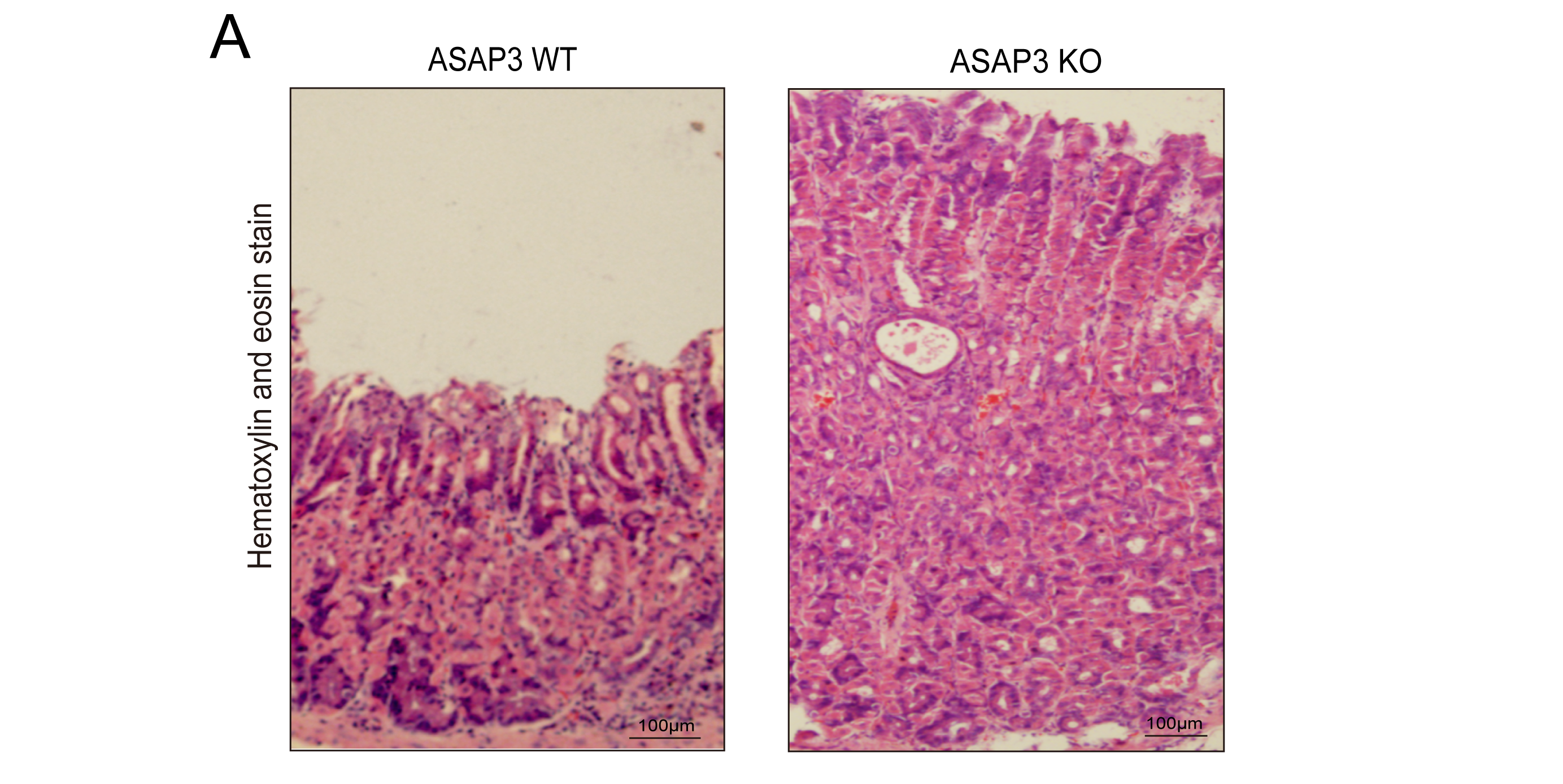


**Supplementary Figure 9.** **Hematoxylin and eosin staining revealed a hypertrophic mucosa in ASAP3-deficient mice.**

Representative Hematoxylin and eosin staining images of gastric mucosa tissue

sections from ASAP3-deficient and WT mice.

**
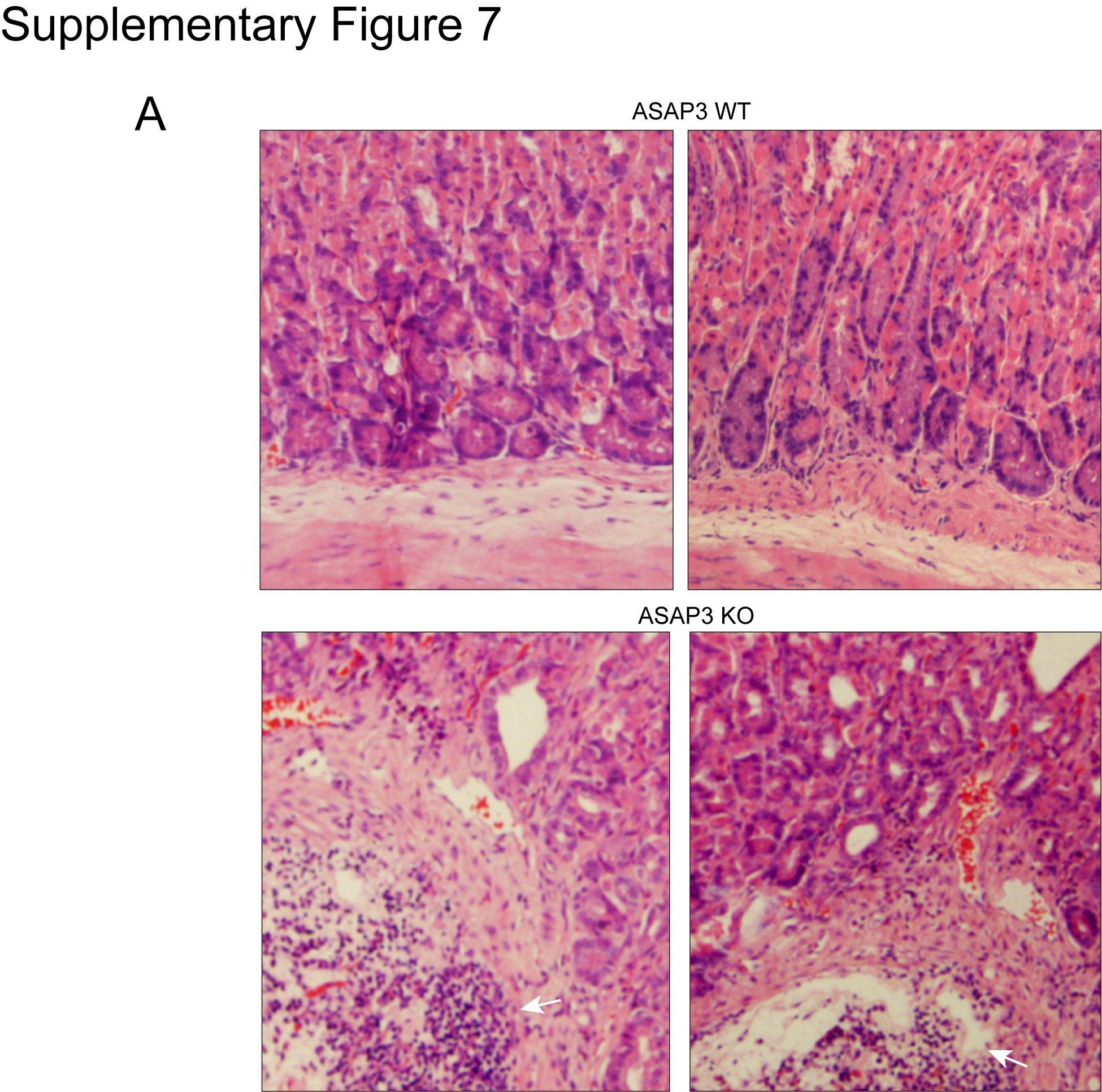
**

**Supplementary Figure 10. Hematoxylin and eosin staining revealed infiltration of inflammatory cells in the mucosa tissue of ASAP3-deficient mice.**

Representative Hematoxylin and eosin staining images of gastric mucosa tissue sections from ASAP3-deficient and WT mice. The white arrows indicate infiltration of inflammatory cells in the mucosa tissue of ASAP3-deficient mice.
